# A Community Detection Algorithm Based on Topology Potential and Spectral Clustering

**DOI:** 10.1155/2014/329325

**Published:** 2014-07-22

**Authors:** Zhixiao Wang, Zhaotong Chen, Ya Zhao, Shaoda Chen

**Affiliations:** School of Computer Science and Technology, China University of Mining and Technology, Xuzhou, Jiangsu 221116, China

## Abstract

Community detection is of great value for complex networks in understanding their inherent law and predicting their behavior. Spectral clustering algorithms have been successfully applied in community detection. This kind of methods has two inadequacies: one is that the input matrixes they used cannot provide sufficient structural information for community detection and the other is that they cannot necessarily derive the proper community number from the ladder distribution of eigenvector elements. In order to solve these problems, this paper puts forward a novel community detection algorithm based on topology potential and spectral clustering. The new algorithm constructs the normalized Laplacian matrix with nodes' topology potential, which contains rich structural information of the network. In addition, the new algorithm can automatically get the optimal community number from the local maximum potential nodes. Experiments results showed that the new algorithm gave excellent performance on artificial networks and real world networks and outperforms other community detection methods.

## 1. Introduction

Most networks show community structure [1]. The results of community detection are meaningful for forecasting the behavior and evolution trend of complex networks [2]. For example, in World Wide Web, community detection can be used to improve the performance of search engine, in social networks, community detection can be used to forecast the information propagation among users [3], in electronic commerce area, community detection can be used to select potential user for advertising; and in bioengineering area, community detection can be used to recognize functions of protein [4].

In recent years, many methods inspired by different paradigms are put forward for community detection [5]. Among these efforts, spectral clustering has shown to be successful [6], for it is very simple to implement and can be solved by standard linear algebra methods.

The traditional spectral clustering methods are based on kinds of input matrixes, such as the adjacency matrix, the standard Laplacian matrix, and the normalized Laplacian matrix. The standard Laplacian matrix is defined as *L* = *D* − *A*, and the normalized Laplacian matrix is defined as *L* = *D* ^−1^ *A*, where *A* is adjacency matrix and *D* is a diagonal matrix with elements *D*_*ii*_ being the degree of the *i*th node.

Almost all above matrixes are constructed with the adjacency matrix and diagonal matrix of networks. These matrixes can only reflect the local relationship between a node and its direct neighbors, as [6] pointed out, “the eigenvalues and eigenvectors of traditional input matrixes cannot provide sufficient structural information for community detection.” As a result, the accuracy of community detection may decrease.

What is more, the community number *k* must be set in advance for the spectral clustering method based on standard Laplacian matrix. The normalized Laplacian matrix can solve this problem to some extent, which has *k* nontrivial eigenvalues close to the biggest eigenvalue 1. The eigenvector elements corresponding to these eigenvalues present ladder distribution. The proper community number of communities can be estimated by the ladders. However, when the community structure of network is not clear, the eigenvector elements cannot show obvious ladder distribution but an approximately continuous curve [7]. In this case, we cannot get the proper community number from the ladder distribution of eigenvector elements.

In order to solve these problems, this paper puts forward a novel community detection algorithm based on topology potential and spectral clustering. The algorithm constructs the normalized Laplacian matrix with topology potential of network nodes. The topology potential of a node is the sum of potential components produced by neighbors at the position of this node. The topology potential describes the complicated interaction among nodes and contains rich structural information of the network. This structural information is meaningful for community detection. In addition, the new algorithm can automatically get the optimal community number from the local maximum potential nodes, whether the community structure of network is obvious or not. Experiments results showed that the new algorithm can improve the accuracy of community detection and has significant adaptability.

This paper is organized as follows.  Section 2 describes related works.  Section 3 introduces the concept of topology potential.  Section 4 shows the new community detection algorithm based on topology potential and spectral clustering.  Section 5 is simulation experiment and results.  Section 6 comes to the conclusion of this paper.

## 2. Related Works

Spectral clustering algorithms have been successfully applied to community detection. From the perspective of input matrix, spectral clustering methods can be divided into the adjacency matrix [8], the standard Laplacian matrix [9], the normalized Laplacian matrix [10], the modularity matrix [11], and the correlation matrix [12]. Reference [13] found that the normalized Laplacian matrix significantly outperforms the other matrixes in identifying the community structure of networks.

In order to improve the performance of spectral clustering, many nontraditional spectral clustering algorithms have been proposed [6], such as complement based spectral clustering [14], complex eigenvector based spectral clustering [15], semisupervised spectral clustering [16], and eigenspace-based spectral clustering [17]. Zarei and Samani [14] gave out a spectral method based on the network complement and anticommunity concept, declaring “the spectrum of matrixes corresponding to a network complement reveals the communities more accurately than that of a matrix corresponding to the network itself.” Zarei et al. [15] also put forward a spectrum method based on complex eigenvectors and found that the complex eigenvectors of network matrixes showed better performance in community detection. Mavroeidis [16] proposed a semisupervised spectral clustering, and its results showed that the partial supervision cannot only improve the quality of spectral clustering but also accelerate the spectral clustering. Ma et al. [17] presented an eigenspace-based spectral method for community detection, which can identify both the overlapping and hierarchical community without increasing the time complexity. All these methods try to integrate some additional topology structure information into input matrixes.

Except for methods mentioned above, there are some other newly developed spectral methods for community detection. Gong et al. [18] proposed a spectral algorithm utilizing multiple eigenvectors to identify the communities in networks, which performed better for more spectral information is used. Newman [19] found that, within the spectral approximations, community detection by modularity maximization, community detection by statistical inference, and normalized-cut graph partitioning are identical. With the large-deviation theory, Bo et al. [20] established a relationship between the hierarchical community structure of a network and the local mixing properties.

Recently, a novel theory-topology potential theory was introduced to complex network for community detection [21]. Because of its inherent advantage in time complexity and performance, this theory has attracted plenty of attention. Gan et al. [21] put forward a topology-potential-based community detection algorithm. With the algorithm, the community structure can be uncovered by “detecting all local high potential areas margined by low potential nodes.” Han et al. [22] proposed an overlapping community detection algorithm, which divides networks into separate communities by “spreading outward from each local maximum potential node.” Zhang et al. [23] proposed a variable scale network overlapping community identification method based on topology potential. In order to identify overlapping nodes, this method defined an identity uncertainty measure related to topology potential. These above topology-potential-based methods show better performance in community detection; however, there is a weakness for almost all these methods; that is, they definitely need additional strategies or parameters to determine the community attachment of nodes, such as the benefit function in [21] and the parameter *ξ* in [23].

Different from above works, this paper puts forward a novel community detection algorithm, which combines spectral clustering and topology potential, making best use of their advantages and bypassing their disadvantages. The new algorithm constructs the normalized Laplacian matrix with topology potential of network nodes. The topology potential contains rich structural information of the network, which is meaningful for community detection. What's more, the new algorithm can automatically judge the optimal community number from the local maximum potential nodes, whether the community structure of complex network is obvious or not.

## 3. Topology Potential

The topology potential field theory is an important branch of the field theory. People abstracted the classical field as a mathematical model to describe noncontact interaction between objects [24]. Any complex network has its relatively stable topology structure; nodes in the network are not isolated, and there exist relationships between nodes linked by edges. Therefore, the topology potential field theory was introduced into complex network to describe the interaction and association among network nodes [22].

Given a network *G* = (*V*, *E*), where *V* = {*υ*_*i*_∣*i* = 1,…, *n*} is a set of nodes, *n* is the total number of nodes, *E* = {(*υ*_*i*_, *υ*_*j*_)∣*υ*_*i*_, *υ*_*j*_ ∈ *V*} is a set of edges. According to the topology potential field theory, the topology potential of any node is defined as follows:

(1) $$\begin{array}{c} \varphi \left(\upsilon_{i}\right)=\overset{k}{\underset{l=1}{\sum }}m\left(\upsilon_{l}\right)\times e^{-{\left(d_{il}/\sigma \right)}^{2}}, \end{array}$$

where *φ*(*υ*_*i*_) is the topology potential of node *υ*_*i*_, 1 ≤ *i* ≤ *n*; the node *υ*_*l*_ is a node within the influence scope of node *υ*_*i*_, and *k* is the total number of nodes within the influence scope, 1 ≤ *k* ≤ *n* − 1, 1 ≤ *l* ≤ *k*; *d*_*il*_ is the hops between s *υ*_*i*_ and *υ*_*l*_; *m*(*υ*_*l*_) is the mass of node *υ*_*l*_; generally speaking, it is set to 1, and the mass difference between nodes is ignored; *σ* is an impact factor used to control the influence scope of node, the maximum scope is $\left\lfloor 3\sigma /\sqrt{2}\right\rfloor$ hops.

The impact factor *σ* will affect topology potential field and the influence scope of node. If *σ* is small, the interaction and association among nodes is weak. And when *σ* → 0, there is even no interaction and association. Conversely, if *σ* is big, the interaction and association become strong, and in extreme conditions, all nodes associate with each. Therefore, we need to select suitable value, so as to make the distribution of topology potential value reflect the structure characteristics of network. Potential entropy has been introduced to evaluate the rationality of topology potential value distribution [21].

Suppose the topology potential of nodes *υ*_1_, *υ*_2_,…, *υ*_*n*_ are *φ*(*υ*_1_), *φ*(*υ*_2_),…, *φ*(*υ*_*n*_), respectively; the potential entropy *H* is defined as

(2) $$\begin{array}{c} H=-\overset{n}{\underset{i=1}{\sum }}\frac{\varphi \left(\upsilon_{i}\right)}{Z}\cdot \log\left(\frac{\varphi \left(\upsilon_{i}\right)}{Z}\right), \end{array}$$

where *n* is the total number of nodes; *Z* = ∑_*i*=1_^*n*^ *φ*(*υ*_*i*_) is a normalization factor. When topology potential field achieves the smallest potential entropy, the impact factor value is optimal [25].

As can be seen from the formula (1), the topology potential of a node totally depends on the topology structure of its surroundings, which reflects the influence ability of another node over it. Obviously, the topology potential contains rich structural information of the network, which offers a desirable solution to the insufficient structural information in the traditional Laplacian matrix. If we construct the Laplacian matrix by using topology potential of network nodes, the additional structural information can be provided for community detection. So, this paper puts forward a novel algorithm based on topology potential and spectral clustering to improve the performance of community detection, and Section 4 will describe the new algorithm in detail.

## 4. Community Detection Algorithm

In this section, we will give out a novel community detection algorithm based on topology potential and spectral clustering. The new algorithm is described as follows.

*Input*: complex network *G* = (*V*, *E*), the corresponding node set *V* = {*υ*_*i*_∣*i* = 1,…, *n*}, edge set *E* = {(*υ*_*i*_, *υ*_*j*_)∣*υ*_*i*_, *υ*_*j*_ ∈ *V*}.

*Output*: a community partition of *G*.

*Algorithm Description*:

1. calculate the topology potential of node with formula (1);
2. search all local maximum potential nodes of *G*. Suppose we find *k* local maximum potential nodes;
3. construct the potential component matrix *P* and topology potential matrix *T* of *G*;
4. compute the normalized Laplacian matrix *L* = *T* ^−1^ *P* of *G*;
5. compute the first *k* eigenvectors *u*_1_,…, *u*_*k*_ of *L*, *k* is the total number of local maximum potential nodes;
6. map all nodes in *V* to *R* ^*k*^ corresponding to eigenvectors *u*_1_,…, *u*_*k*_;
7. cluster the nodes in *R* ^*k*^ with the *k*-means algorithm into communities *C*_1_,…, *C*_*k*_.

Compared with the traditional spectral clustering method, the new algorithm constructs the normalized Laplacian matrix with the topology potential of nodes and can automatically get the optimal community number from the local maximum potential node.

The following part of the section will focus on the normalized Laplacian matrix construction and local maximum potential node search of the new community detection algorithm.

### 4.1. Normalized Laplacian Matrix Construction

In order to add additional structural information of networks, the normalized Laplacian matrix *L* is redefined as follows:

(3) $$\begin{array}{c} L=T^{-1}P, \end{array}$$

where the adjacency matrix *A* used in the conventional normalized Laplacian matrix is replaced by the potential component matrix *P* and the degree matrix *D* by the topology potential matrix *T*.

The topology potential matrix *T* is an *n*-dimensional diagonal matrix, and the diagonal element *t*_*i*,*i*_ = *φ*(*υ*_*i*_), that is, the topology potential of node *υ*_*i*_.

The potential component matrix *P* is *n* × *n* matrix, and the matrix elements *p*_*i*,*j*_ are the potential component produced by node *υ*_*j*_ at the position node *υ*_*i*_, which is defined as follows:

(4) $$\begin{array}{c} p_{i,j}=m\left(\upsilon_{j}\right)\times e^{-{\left(d_{ij}/\sigma \right)}^{2}},\;1\leq i,j\leq n, \end{array}$$

where *m*(*υ*_*j*_) is the mass of node; *d*_*ij*_ is the hops between node *υ*_*j*_ and node *υ*_*i*_; *σ* is an impact factor used to control the influence scope of node. If *i* = *j*, then *p*_*i*,*j*_ = 0; if node *υ*_*i*_ is out of the influence scope ($\left\lfloor 3\sigma /\sqrt{2}\right\rfloor$) of node *υ*_*j*_, then *p*_*i*,*j*_ = 0.

**Figure 1:**
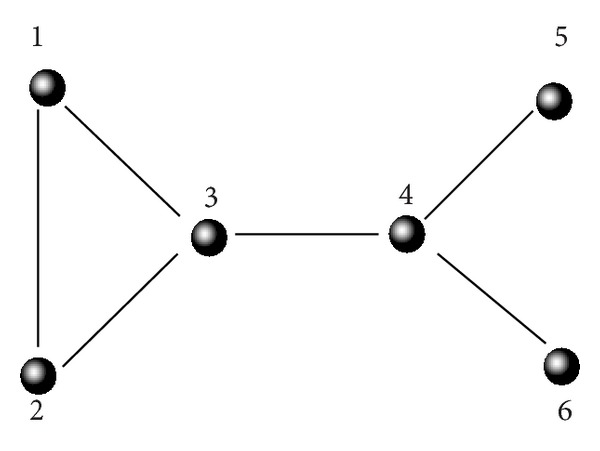
A simple network model.

Figure 1 shows a simple network model, which contains only six nodes. Here, we take the figure as an example to show the construction of the potential component matrix *P* and topology potential matrix *T*. For this network, the selected optimal impact factor *σ* = 1.39; thus, the influence scope of node $\left\lfloor 3\sigma /\sqrt{2}\right\rfloor =2$. We can use formula (1) to get the topology potential of all the six nodes. The topology potential of node 1 is 1.3181, and the topology potentials of the other five nodes are 1.3181, 2.0402, 2.0402, 0.8413, and 0.8413, respectively. Thus, we can get the topology potential matrix *T* of Figure 1:

(5) T=(1.31811.31812.04022.04020.84130.8413).

The topology potential of node 1 is the summation of potential component produced by node 2 (0.5960), node 3 (0.5950), and node 4 (0.1261). Similarly, the topology potential of node 3 is the summation of potential component produced by node 1 (0.5960), node 2 (0.5960), node 4 (0.5950), node 5 (0.1261), and node 6 (0.1261). Thus, we can get the potential component matrix *P*:

(6) $$\begin{array}{c} P=\left(\begin{array}{cccccc} 0 & 0.5960 & 0.5960 & 0.1261 & 0 & 0\\ 0.5960 & 0 & 0.5960 & 0.1261 & 0 & 0\\ 0.5960 & 0.5960 & 0 & 0.5960 & 0.1261 & 0.1261\\ 0.1261 & 0.1261 & 0.5960 & 0 & 0.5960 & 0.5960\\ 0 & 0 & 0.1261 & 0.5960 & 0 & 0.1261\\ 0 & 0 & 0.1261 & 0.5960 & 0.1261 & 0 \end{array}\right). \end{array}$$

Based on formula (2), the normalized Laplacian matrix *L* is

(7) L=T−1P=(00.45210.45210.0957000.452100.45210.0957000.29210.292100.29210.06180.06180.06180.06180.2921000.2921000.14870.702600.1487000.14870.70260.14870).

### 4.2. Local Maximum Potential Node Search

The hill-climbing method is a traditional algorithm for local maximum point search, which may leave out some local maximum points, and search performance is greatly influenced by initial point selection. We give out a new local maximum potential node search algorithm with review to local maximum potential nodes' characteristics.

The key steps of the new search algorithm are shown as follows.

1. All network nodes are initialized to “unvisited.”
2. Randomly choose an “unvisited” node *υ*_*i*_ and compare the topology potential of *υ*_*i*_ with its neighbors'. If the topology potential of *υ*_*i*_ is higher than all neighbors', then jump to step (3); otherwise, jump to step (4).
3. Add *υ*_*i*_ to the local maximum potential node set *K* and mark *υ*_*i*_ as well as its all neighbors “visited.”
4. Mark *υ*_*i*_ “visited,” and mark neighbors with lower topology potential than *υ*_*i*_'s “visited.”
5. Repeat steps (2), (3), and (4), until all nodes in network are marked “visited.”
6. If there are two local maximum potential nodes whose distance, that is, hops, is smaller than $\left\lfloor 3\sigma /\sqrt{2}\right\rfloor$, then we delete the smaller one from *K*.
7. Output the final local maximum potential node set *K*.

More details about local maximum potential node search can be referred to [24].

## 5. Simulation Experiments

In this section, a series of experiments will be carried out to empirically evaluate the performance of the new algorithm. Simulation program was implemented with MATLAB. The experiment data include two kinds of complex networks: artificial networks and real world networks. The artificial networks were generated by ad hoc model [26] and LFR Benchmark generator [27]. LFR Benchmark is a network generator, which produces networks with power-law degree distribution and with implanted communities within the network [27]. The real world networks come from http://www-personal.umich.edu/~mejn/netdata/. The normalized mutual information (NMI) [28], a widely used measure, is calculated for the community partition by each algorithm.

### 5.1. Ad Hoc Network

The generated ad hoc network, with 128 nodes, is split into 4 communities containing 32 nodes each. The parameter *z*_out_ is the average edge that links one node with other nodes of different communities. As *z*_out_ increases, the community structure of the ad hoc network becomes ambiguous gradually. In the experiment, we changed *z*_out_ from 0 to 8 and observed the corresponding NMI produced by six methods: our algorithm, traditional spectral method, the *k*-means based on diffusion distance (DD *k*-means) [26], the *k*-means based on dissimilarity index (DI *k*-means) [26], Fast Newman algorithm [29], and Extremal Optimization method [30].

**Figure 2:**
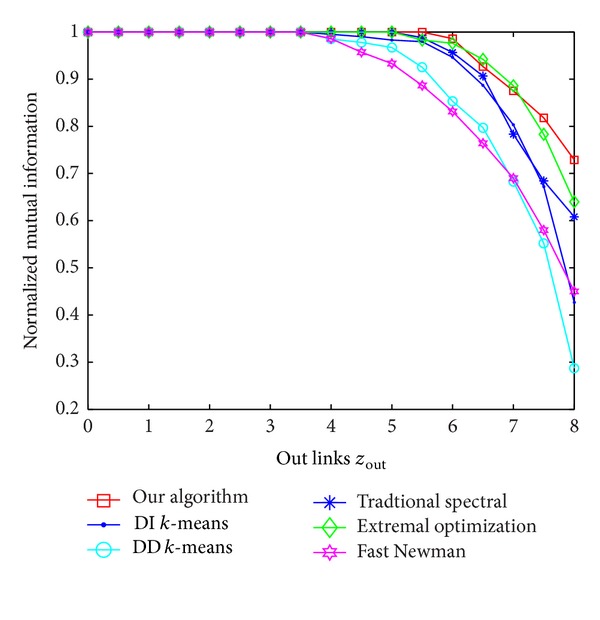
The NMI of the six methods with the change of *z*_out_.

The experiment results are shown in Figure 2, where *y*-axis represents the value of NMI, and each point represents an average 30 simulation experiments. Compared with the other five methods, our algorithm is only slightly worse than the Extremal Optimization method for 6.4 < *z*_out_ < 7.1. Our algorithm has a good performance for the ad hoc network, and the accurate rate is more than 98% for *z*_out_ < 5.5.

### 5.2. LFR Network

In generated LFR networks, the node degree and community size distribute according to power law. A mixing parameter *μ* is defined as the ratio between the external degree of a node with respect to its community and the total degree of the node [26], 0 ≤ *μ* ≤ 1. As *μ* increases, the community structure of the LFR network becomes ambiguous gradually. There are many other parameters used to control the generated LFR networks: the number of nodes *N*, the average node degree *k*, the maximum node degree max_*k*_, the minimum community size min⁡_*c*_, and the maximum community size max⁡_*c*_ [26].

In our experiments, we changed *μ* from 0 to 0.8 and observed the corresponding NMI produced by seven methods: our algorithm, traditional spectral method, Danon algorithm, Louvain algorithm, Infomap algorithm, Clique Percolation algorithm [28], and Fast Newman algorithm [29]. We used the default parameter configuration where *N* = 1000, *k* = 15, max⁡_*k*_ = 50, min⁡_*c*_ = 20, and max⁡_*c*_ = 50.

**Figure 3:**
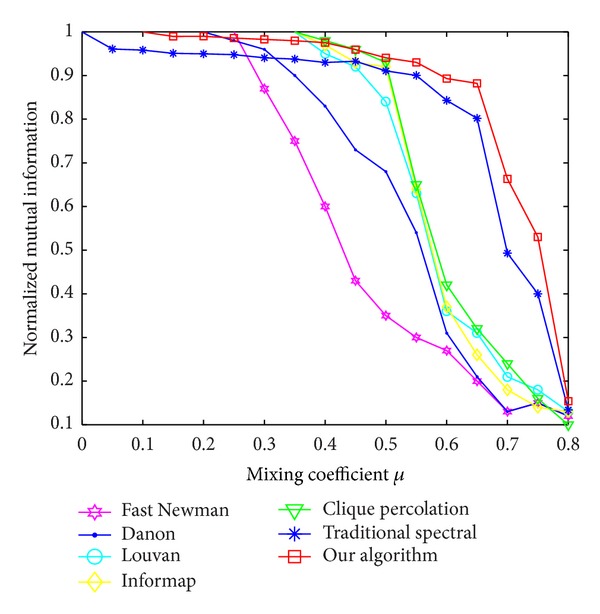
The NMI of the seven methods with the change of *μ*.

The experiment results are shown in Figure 3, where *y*-axis represents the value of NMI. Compared with other six algorithms, our algorithm performs quite well, and its accuracy is only slightly worse than that of the Clique Percolation, Louvan, and Informap in the case of 0.25 < *μ* < 0.45. Because of the complexity of topology potential distribution in the topology potential field, local maximum potential nodes may not necessarily the real central nodes of communities in some cases, resulting in the split or merger of some actual communities and the fluctuation of NMI value.

### 5.3. American College Football Network

The American College Football network [18] contains 115 teams, among which 616 games were carried out. In the network, nodes represent teams and edges games. All teams are organized into 12 conferences, and each of which contains about 8–12 teams. These 12 conferences are Atlantic Coast, Big East, Big Ten, Big Twelve, Conference USA, Independents, Mid American, Mountain West, Pacific Ten, Southeastern, Sun Belt, and Western Athletic.

We compared our algorithm with other three algorithms, including the traditional spectral algorithm, the spectral algorithm based on modularity *Q* [18], and CMITP (community members identification based on topology potential) [22].

**Figure 4:**
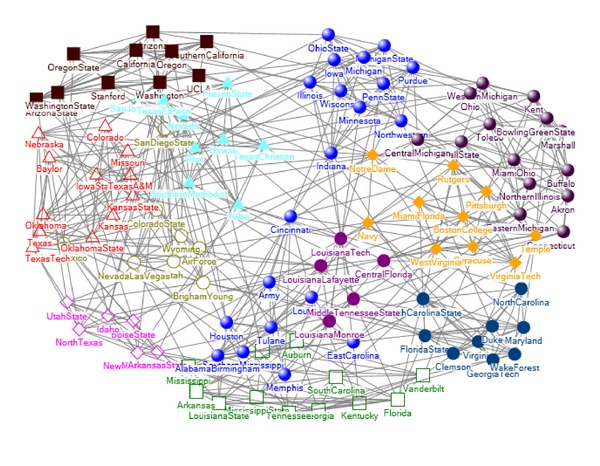
The community detection results by our algorithm.

**Figure 5:**
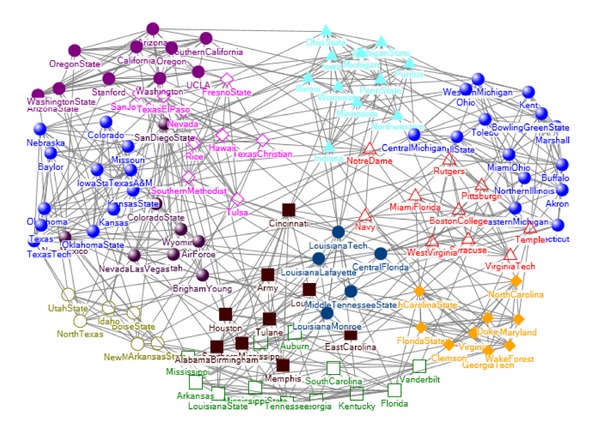
The community detection results by the traditional spectral algorithm.

Firstly, we compared our algorithm with the traditional spectral algorithm. The latter cannot obtain the football network community number from the ladder distribution of eigenvector elements; therefore, we set its community number the same as our method. Figures 4 and 5 show the community detection results by our algorithm and traditional spectral algorithm, respectively. Each node represents a competing team, using its name as label. The teams in same community are marked the same color. For this network, the traditional spectral algorithm gets six correct communities: Mountain West, Atlantic Coast, Southeastern, Pacific Ten, Big Ten, and Conference USA. Compared with the traditional spectral algorithm, our algorithm gets three new correct communities: Big Twelve, Big East, and Mid American. For the conference Western Atlantic, our algorithm gets 9 correct teams, with only 1 team missing. Both algorithms split the conference Sun Belt and Independents.

Secondly, we compared our algorithm with the spectral algorithm based on modularity. Tables 1 and 2 show the results of our algorithm and the spectral algorithm based on modularity, respectively. The conference names are listed in the leftmost column, and columns *a* ~ *k* represent the communities found by the two algorithms. Each found community consists of teams from one or more conferences as indicated by the numbers in the corresponding column [18]. The spectral algorithm based on modularity divided this network into 10 communities, and six communities are correctly detected: Atlantic Coast, Big East, Big Ten, Big Twelve, Mid American, and Pacific Ten. Compared with the spectral algorithm based on modularity, our algorithm found 11 communities and got a new correct communities Mountain West.

The CMITP method divided this network into 17 communities, and there are many overlapping nodes between communities, such as nodes “Hawaii” and “Nevada.” Table 3 shows the community number, *Q* and NMI of four different algorithms. Compared with other three methods, our algorithm got the highest NMI 0.9292. In addition, our algorithm found 11 communities, which is the closest to the real community number 12.

### 5.4. The Influence of Impact Factor *σ* on Algorithm Performance

**Figure 6:**
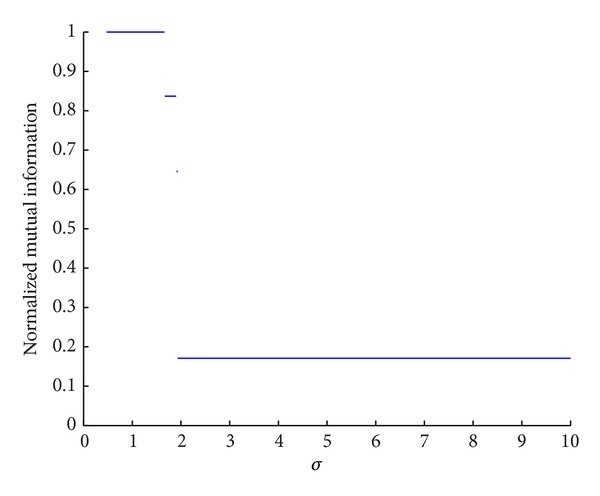
The influence of *σ* on algorithm performance.

The impact factor *σ* will affect topology potential field and the influence scope of node. With different impact factor *σ*, the distribution of topology potential value will be different. These changes may bring out different community detecting results. We take a real world network, the Zachary karate club network, to analyze the influence of impact factor *σ* on algorithm performance.  Figure 6 shows the NMI of our algorithm with different impact factor *σ*.

Figure 6 shows that if *σ* ≤ 0.4716, the NMI is 0; if 0.4716 < *σ* ≤ 1.66, the NMI is 1; if 1.66 < *σ* ≤ 1.90, the NMI is 0.8372; if 1.90 < *σ* ≤ 1.934, the NMI is 0.6459; if *σ* > 1.934, the NMI is 0.1701. The analysis is as follows. The maximum influence scope of node is $\left\lfloor 3\sigma /\sqrt{2}\right\rfloor$ hops. When *σ* ≤ 0.4716, the influence scope of node $\left\lfloor 3\sigma /\sqrt{2}\right\rfloor$ = 0; it means that all nodes are isolated and have same topology potential value. For Zachary network, the optimal *σ* is 1.02 according to formula (2). When 0.4716 < *σ* ≤ 1.66, we can detect accurate community structure, and the NMI is 1. But as *σ* further increases, one node can associate with almost all the other nodes. In this case, the distribution of topology potential value cannot truly reflect the structure characteristics of network; therefore, the community detecting results are bad. In a word, as long as the impact factor *σ* is set near the optimal value, our algorithm can get good outcomes.

## 6. Conclusion

Identifying community structure is crucial for understanding complex networks. Recently, spectral clustering algorithms have been successfully applied in community detection. The traditional spectral clustering methods cannot provide sufficient structural information for community detection and cannot always get the community number from the ladder distribution of eigenvector elements. Aiming at these inadequacies, this paper puts forward a novel community detection algorithm based on topology potential and spectral clustering. The new algorithm constructs the normalized Laplacian matrix with network nodes' topology potential, which contains rich structural information of the network. In addition, the new algorithm can automatically judge the optimal community number from the local maximum potential nodes. Experiments on ad hoc network, LFR network, and the American college football network showed that the new algorithm can improve the accuracy of community detection and has significant adaptability.

## Figures and Tables

**Table 1:** The community detection results of our algorithm.

	*a*	*b*	*c*	*d*	*e*	*f*	*g*	*h*	*i*	*j*	*k*	
Atlantic Coast					9							9
Big East						8						8
Big Ten		11										11
Big Twelve									12			12
Conference USA		9					1					10
Independents				1		2	1			1		5
Mid American				13								13
Mountain West											8	8
Pac Ten								10				10
Southeastern			12									12
Sun Belt							3			4		7
Western Atlantic	9									1		10
	**9**	**20**	**12**	**14**	**9**	**10**	**5**	**10**	**12**	**6**	**8**	**115**

**Table 2:** The community detection results of the spectral algorithm based on modularity.

	*a*	*b*	*c*	*d*	*e*	*f*	*g*	*h*	*i*	*j*	
Atlantic Coast							9				9
Big East				8							8
Big Ten		11									11
Big Twelve			12								12
Conference USA						1			9		10
Independents				2				2		1	5
Mid American								13			13
Mountain West										8	8
Pacific Ten	10										10
Southeastern					12						12
Sunbelt					3					4	7
Western Atlantic					1	8				1	10
	**10**	**11**	**12**	**10**	**16**	**9**	**9**	**15**	**9**	**14**	**115**

**Table 3:** The community number, *Q* and NMI of four algorithms.

	Community number	*Q*	NMI
The real community	12	0.5540	1.0000
Our algorithm	11	0.5879	0.9292
Traditional spectral	11	0.5792	0.8879
Spectral based on modularity	10	0.5870	0.8800
CMITP	17	0.5538	—
